# l-Serine and EPA Relieve Chronic Low-Back and Knee Pain in Adults: A Randomized, Double-Blind, Placebo-Controlled Trial

**DOI:** 10.1093/jn/nxaa156

**Published:** 2020-06-10

**Authors:** Ikuko Sasahara, Akiko Yamamoto, Masamichi Takeshita, Yasuyo Suga, Katsuya Suzuki, Natsumi Nishikata, Michihiro Takada, Masaki Hashimoto, Tomoyuki Mine, Yasuo Kobuna, Kenji Nagao

**Affiliations:** Institute of Food Sciences and Technologies, Ajinomoto Co, Inc, Kawasaki, Kanagawa, Japan; Institute of Food Sciences and Technologies, Ajinomoto Co, Inc, Kawasaki, Kanagawa, Japan; Research Institute for Bioscience Products & Fine Chemicals, Ajinomoto Co, Inc, Kawasaki, Kanagawa, Japan; Institute of Food Sciences and Technologies, Ajinomoto Co, Inc, Kawasaki, Kanagawa, Japan; Institute of Food Sciences and Technologies, Ajinomoto Co, Inc, Kawasaki, Kanagawa, Japan; Research Institute for Bioscience Products & Fine Chemicals, Ajinomoto Co, Inc, Kawasaki, Kanagawa, Japan; Research Institute for Bioscience Products & Fine Chemicals, Ajinomoto Co, Inc, Kawasaki, Kanagawa, Japan; Direct Marketing Department, Ajinomoto Co, Inc, Tokyo, Japan; Institute of Food Sciences and Technologies, Ajinomoto Co, Inc, Kawasaki, Kanagawa, Japan; Kobuna Orthopedic Clinic, Maebashi, Gunma, Japan; Research Institute for Bioscience Products & Fine Chemicals, Ajinomoto Co, Inc, Kawasaki, Kanagawa, Japan

**Keywords:** multiple site pain, neuropathic pain, low-back and knee pain, clinical, Japan Low Back Pain Evaluation Questionnaire (JLEQ), Japanese Knee Osteoarthritis Measure (JKOM), Brief Pain Inventory (BPI)

## Abstract

**Background:**

Multisite pain, including low-back and knee pain, is a major health issue that greatly decreases quality of life.

**Objectives:**

This study analyzed the effects of l-serine, which provides necessary components for nerve function, and EPA, which exerts anti-inflammatory properties, on pain scores of adults with pain in at least the low back and knee for ≥3 mo.

**Methods:**

This was a randomized, double-blind, placebo-controlled, parallel-group study. The Japan Low Back Pain Evaluation Questionnaire (JLEQ) and Japanese Knee Osteoarthritis Measure (JKOM) were applied as primary outcomes. The Brief Pain Inventory (BPI) and safety evaluation were secondary outcomes. We enrolled 120 participants aged ≥20 y (36 men and 84 women: mean ± SD age = 40.8 ± 10.9 y). The participants were randomly allocated to either the active group (daily ingestion of 594 mg l-serine and 149 mg EPA) or placebo group. The study period consisted of 8-wk dosing and 4-wk posttreatment observation. ANCOVA between groups for each time point was conducted using the baseline scores as covariates.

**Results:**

The JLEQ scores (active compared with placebo: 14.2 ± 11.2 compared with 19.0 ± 10.2) at week 8 were lower in the active group (*P* < 0.001). The JKOM scores at week 4 (11.7 ± 9.0 compared with 13.9 ± 7.9), week 8 (10.4 ± 7.9 compared with 13.1 ± 7.1), and week 12 (10.3 ± 7.4 compared with 13.8 ± 7.5) were lower in the active group (*P* ≤ 0.04). Additionally, the active group had 11–27% better scores compared with the placebo group for BPI1 (worst pain), BPI3 (average pain), and BPI5D (pain during moving) at week 4 (*P* ≤ 0.028) and week 8 (*P* ≤ 0.019), respectively, and BPI5D was 23% better in the active group at week 12 (*P* = 0.007). No adverse events were observed.

**Conclusions:**

l-Serine and EPA were effective for pain relief in adults with low-back and knee pain after multiplicity adjustment.

This trial was registered at the University Hospital Medical Information Network Clinical Trials Registry as UMIN000035056.

## Introduction

Pain often occurs concurrently at multiple sites, such as the lower back, neck, shoulders, hip, and knee area (1). Low-back pain (LBP) and knee pain constitute major health issues in the adult population (2). Up to 41.4% of the Japanese adult population experiences musculoskeletal pain, with the lower back being the common site of pain for both sexes (3). This study demonstrated that the neck and shoulder area showed the highest prevalence of pain (20.3%), followed by the lower back area (19.1%), and the hip and knee areas (11.1%). A community-based survey targeting >4000 participants in Japan demonstrated that the number of people with these types of pain increases with age (4). Such pain is associated with daily activity impairment as well as loss of work productivity, which significantly impacts quality of life (QOL) (1, 5). In a survey of Japanese adults and the UK Biobank study, greater pain severity and higher number of pain sites were associated with higher presenteeism (6, 7). Another study demonstrated that the total incremental cost of health care due to pain was $635 billion in the United States (8). Current care for LBP, such as nonsteroidal anti-inflammatory drugs, only offers temporary relief with limited effectiveness and poses a high risk of gastrointestinal side effects (5).

Similarly, knee pain is a common complaint experienced by people of all ages. It has been estimated that ≥25% of the elderly population has chronic knee pain, defined as pain occurring on most days of a recent month (9). Knee pain affects daily life and reduces mobility, which can progress to further disabling symptoms. Glucosamine and chondroitin are popular supplements that are commercially available worldwide, but purported benefits have not been consistently supported in the literature (10). Thus, interventions to effectively manage such chronic pain are sought.

l-Serine (l-Ser) is an amino acid that is essential for maintaining normal functions of the nervous system. It is a precursor for the synthesis of phosphoglycerols and complex macromolecules such as sphingolipids and glycolipids, which are important membrane components and myelin constituents (11). When neuronal cells are cultured under serine-deficient conditions, the concentrations of phosphatidylserine and sphingolipids decrease. Demyelination contributes to the development of neuropathic pain by disrupting the molecular and structural features of nerve fibers (12). Patients with hereditary l-Ser deficiency are reported to have polyneuropathy (13). Additionally, there is a report that the l-Ser concentration in blood decreases with age (14). These findings indicate the importance of l-Ser for maintaining normal function of the nervous system, which could be linked to providing beneficial support in chronic pain management.

Furthermore, numerous studies have found that dietary supplementation with ω-3 PUFAs, mainly as combinations of EPA and DHA, is efficacious in reducing joint swelling and pain, morning stiffness, and nonsteroidal anti-inflammatory drug usage in rheumatoid arthritis patients (15, 16). EPA, an ω-3 lipid, exerts anti-inflammatory properties after being metabolized to the anti-inflammatory lipid mediator resolvin E1. Metabolites such as resolvin E1 compete with metabolites from ω-6 PUFAs to promote the resolution of the inflammatory cycle and have been increasingly recognized as important players in the attenuation of inflammation and regulation of autoimmunity (17, 18). Resolvin E1 is reported to alleviate pain and hyperalgesia in response to heat and mechanical stimuli (19–22), and findings indicate that resolvin E1 abolishes TNF-α–evoked *N*-methyl-d-aspartic acid (NMDA) receptor hyperactivity in spinal dorsal horn neurons, thereby normalizing the spinal synaptic plasticity that has been implicated in generating pain hypersensitivity (21). These reports suggest that EPA supplementation can be beneficial for chronic pain induced by inflammation.

In particular, localized inflammation of the dorsal root ganglion (DRG), which extends its axons to the peripheral nerves, has been proposed to play an important role in neuropathic pain. Inflammatory processes within the DRG per se change excitability of the DRG neurons (23). Early work has demonstrated the importance of the ω-3 PUFAs, EPA and DHA, in attenuating inflammation in the DRG (24). Also, interestingly, a previous study has shown that the l-Ser biosynthesis system in the DRG is affected in a nonclinical model of painful peripheral neuropathy (25). The authors demonstrated that the localized expression of l-Ser biosynthesis enzyme in satellite cells, and not in neuronal cell bodies, plays an important role, and reported decreased l-Ser biosynthesis in the DRG in a paclitaxel-induced hyperalgesia model. Administration of l-Ser improved peripheral hyperalgesia and improved sensory nerve conduction velocity in this model. Based on the above findings, we hypothesized that the combination of l-Ser, which provides necessary components for maintaining nerve function, and EPA, which exerts anti-inflammatory properties, could synergistically alleviate chronic pain, especially in the DRG. In this study, we targeted the generally healthy adult population that experiences pain in multiple body sites. The study aimed to determine the effects of l-Ser and EPA on the pain scores of participants with pain in at least the low back and knee for ≥3 mo. The study was a randomized, double-blind, placebo-controlled, parallel-group design and used multiple validated measures for evaluating LBP and knee pain.

## Methods

### Trial design

This study was an 8-wk randomized, double-blind, placebo-controlled, parallel-group study followed by a 4-wk posttreatment observation period.

### Ethics statements

This study was conducted in accordance with the Declaration of Helsinki. All the participants were informed of the nature of the experimental procedure before written informed consent was obtained. The study was approved by the ethics committee of Kobuna Orthopedic Surgery and Ajinomoto Co, Inc, and was registered at the University Hospital Medical Information Network Clinical Trials Registry as UMIN000035056.

### Inclusion and exclusion criteria

We enrolled a generally healthy adult population. Study participants were considered eligible if they met the following criteria: *1*) aged ≥20 y; and *2*) having pain in at least the low back and knee for ≥3 mo based on a PainDETECT score of 13–38, which includes pain with neuropathic components, ranging from a mixed phenotype of nociceptive and neuropathic pain as well as pain highly indicative as being neuropathic (26). Exclusion criteria included: *1*) having nociceptive pain, including wounds, burn injury, and bruising; *2*) taking constant medication that affects pain; *3*) having clear causes of pain, such as hernia, spinal canal stenosis, or knee osteoarthropathy; *4*) having a history of surgery for the same pain in the past; *5*) having psychiatric problems as assessed by the Brief Scale for Psychiatric Problems in Orthopaedic Patients; *6*) taking functional food or supplements that could influence the outcome of the study; *7*) taking amino acid, protein, or EPA supplements; *8*) being pregnant or lactating; and *9*) having allergies to fish or soy food.

### Study design, randomization, and blinding

Sample size was calculated considering type 1 (α) error and type 2 (β) error based on the statistical significance test of the primary end point. Referring to the internal pilot single-arm open-label study, participants who had neuropathic LBP exhibited improvement of the pain score assessed by a 10-point scale as a mean of 2.1 ± 1.6 (*n* = 31) after ingesting the l-Ser and EPA for 8 wk. In addition, participants who had neuropathic knee pain exhibited similar improvement by a mean of 2.1 ± 1.5 (*n* = 36). Based on these results, the mean effect sizes on neuropathic LBP and knee pain were both estimated as 2.1, the SDs were conservatively estimated as 1.81, and for the placebo group, the mean effect size was estimated as 1.0 (27), and the SD was equally set to 1.81. Under these settings, the fixed sequence procedure was used, that is, the score for LBP was first tested by the *t* test, and then the score for knee pain was tested by the *t* test only when a significant difference was observed for LBP. As a result, when 58 participants were included in each treatment group, the power (1 − β) = 90.1% for LBP and 81.2% for knee pain at the α = 0.05 level for the entire procedure. Considering dropouts in each group, the final sample size was determined to be 60 participants in each group.

**FIGURE 1:**
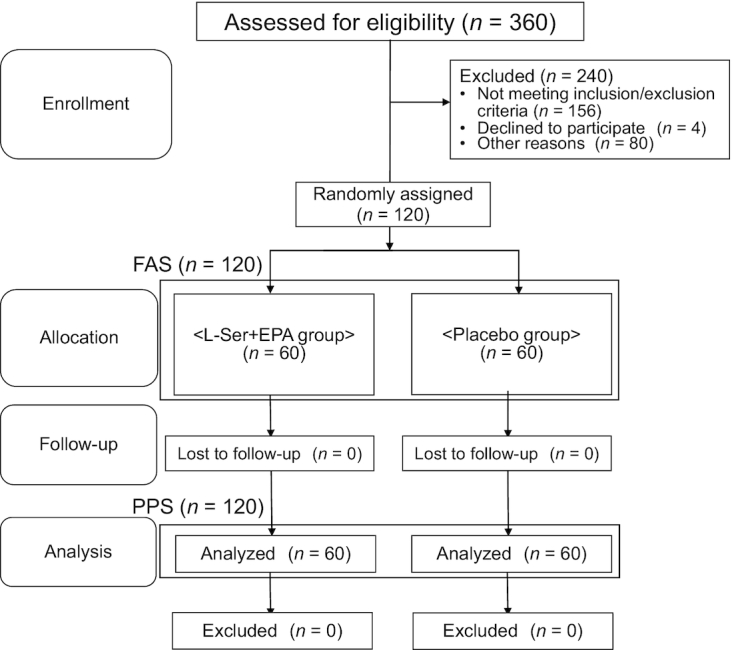
CONSORT diagram for study recruitment. Flow diagram of enrollment and allocation to either the l-serine (l-Ser) + EPA supplementation group or the placebo group of the study. FAS, full analysis set; PPS, per protocol set.

There were 360 applicants, and 120 participants were included in the study (**Figure 1**). We randomly allocated participants to either an l-Ser + EPA supplementation group or a placebo group, and afterwards checked that the following variables were equally distributed in both groups: sex, the Japan Low Back Pain Evaluation Questionnaire (JLEQ) score, and the Japanese Knee Osteoarthritis Measure (JKOM) score. Randomization and allocation were concealed from the researchers, clinicians at the medical institutions, and participants until the final analyses were completed. The allocation table was sealed and stored until key opening by an independent controller.

Participants were provided with either l-Ser + EPA supplementation or placebo, 4 capsules/d. To eliminate the effects of eating meals, they were instructed to ingest the capsules ≥2 h after dinner, before going to bed. Both the active sample and placebo were encapsulated in a soft vegetable film capsule. One l-Ser + EPA capsule contained 148.4 mg l-Ser and 148.4 mg purified fish oil, which contained 37.2 mg EPA, 18 mg beeswax, and 9.5 mg soy lecithin on average. One placebo capsule contained 148.4 mg dextrin, 148.4 mg safflower oil, 18 mg beeswax, and 9.5 mg soy lecithin on average. The total daily intake in the active group was 594 mg l-Ser and 149 mg EPA. Twenty-eight capsules were packaged in an aluminum pouch, corresponding to supplementation for 1 wk. These capsules were manufactured by Sunsho Pharmaceutical Co Ltd.

**FIGURE 2:**
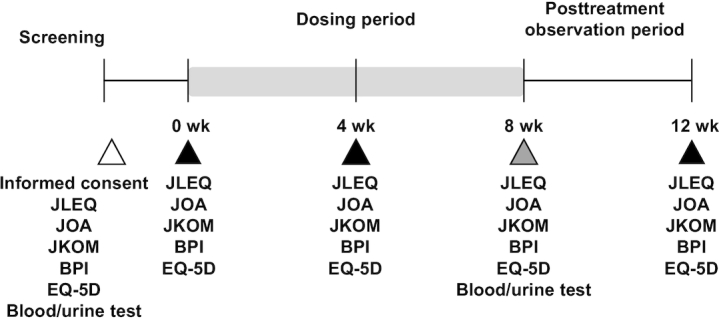
Study flow and outcome measurements during 8 wk of the dosing period and 4 wk of the posttreatment observation period. Scoring on the Japan Low Back Pain Evaluation Questionnaire (JLEQ), Japanese Knee Osteoarthritis Measure (JKOM), Japanese Orthopedic Association (JOA), and Brief Pain Inventory (BPI), quality-of-life (QOL) assessment by EuroQOL 5 Dimensions 5-level (EQ-5D-5L), and medical interviews were conducted at baseline and weeks 4 and 8 (dosing period), and week 12 (posttreatment period).

The study period consisted of 8 wk of the dosing period, and 4 wk of the posttreatment observation period (**Figure 2**). During the posttreatment period, the participants were instructed to continue with the same lifestyle behavior but to stop ingestion of all study capsules, including both active and placebo. The compliance was confirmed by collecting empty aluminum pouches used for packaging active or placebo capsules after the trial. The primary outcomes of the study included JLEQ and Japanese Orthopedic Association (JOA) scores for LBP, and the JKOM score for knee pain. The secondary outcomes included pain assessment by the Brief Pain Inventory (BPI), QOL assessment by EuroQOL 5 Dimensions 5-level (EQ-5D-5L), and a safety evaluation performed by a clinician based on the blood and urine laboratory test results. JLEQ, JKOM, JOA, BPI, EQ-5D-5L, and medical interviews were conducted at baseline and weeks 4 and 8 (dosing period), and week 12 (posttreatment period). The medical interviews included inquiry about the following items: age, gender, medical history, and allergy history (only at baseline), the condition of participants, and general health issues (at each visit).

### Outcome measurement

#### LBP evaluation

To evaluate the LBP, JLEQ and JOA were used. JLEQ is a self-administered, disease-specific measure for assessing the extent of LBP (28, 29). It consists of the visual analog scale (VAS) for the degree of LBP, the JLEQ-I, and a total of 30 questions. These questions include 7 questions regarding LBP related to activities of daily living over the last several days (JLEQ-II), 17 questions regarding problems due to LBP over the last several days (JLEQ-III), and 6 questions regarding health and psychological condition in the last month (JLEQ-IV). These 30 questions in the latter 3 domains are each ranked on a 5-point scale from no impairment (0 points) to serious impairment (4 points) and then added to produce a total score (maximum 120 points). The total score and scores for each domain (JLEQ-I through -IV) were compared between the active and placebo groups.

The JOA score is used to evaluate the therapeutic outcome of LBP. It is a disease-specific measure for assessing the intensity of LBP from the clinician's point of view (30). It consists of 14 questions with a 3–4-point scale, including 3 questions regarding subjective symptoms, 3 questions regarding objective responses, 7 questions regarding activities of daily living, and 1 question regarding bladder function. These 14 questions in the 4 domains are ranked on the 3–4-point scale and then added according to the designated method to produce a total score (maximum: 29 points; minimum: 6 points) (30).

#### Knee pain evaluation

To evaluate knee pain, JKOM was used. It is a self-administered, disease-specific measure for assessing the extent of knee pain and discomfort (31). It consists of a VAS for the degree of knee pain, JKOM-I, and a total of 25 questions. These questions include 8 questions regarding pain and stiffness in the knee over the last several days (JKOM-II), 10 questions regarding problems in daily life due to knee pain over the last several days (JKOM-III), 5 questions regarding usual activities in the last month (JKOM-IV), and 2 questions regarding general health status in the last month (JKOM-V). These 25 questions in the latter 4 domains are ranked on a 5-point scale from no impairment (0 points) to serious impairment (4 points) and then added to produce a total score (maximum 100 points). The total score and scores for each domain (JKOM-I through -V) were compared between the active and placebo groups.

#### Overall pain assessment and QOL assessment

The BPI was used to evaluate the intensity of the overall pain and the impact of the pain on daily life; it consists of 8 questions (32–34). These 8 questions are ranked on an 11-point scale from no impairment (0 points) to serious impairment (10 points).

The EQ-5D-5L was used to evaluate health-related QOL, and consists of 5 questions. These 5 questions are ranked on a 5-point scale from no impairment (1 point) to serious impairment (5 points) and then analyzed according to the reported method (35).

### Safety evaluation

Systolic and diastolic blood pressure and pulse rate measurements were conducted at baseline and weeks 4, 8, and 12. At baseline and week 8, blood was collected under fasting conditions in the morning. The following blood parameters were measured: white blood cell (WBC) count, RBC count, hemoglobin (Hb), hematocrit (Ht), platelet count, total protein, albumin, aspartate aminotransferase, alanine aminotransferase, lactate dehydrogenase, total bilirubin, alkaline phosphatase, γ-glutamyl transpeptidase, urea nitrogen, creatinine, uric acid, sodium, chlorine, potassium, total cholesterol, LDL cholesterol, HDL cholesterol, triglyceride, fasting plasma glucose concentration (FPG), and glycated hemoglobin (HbA1c). The urine samples were obtained at baseline and week 8, and the following urine parameters were measured: urine protein, urine glucose, and urine occult blood. All plasma biochemical variables were measured by LSI Medience Corporation using automatic analyzer LST-008α (Hitachi High-Tech Corp) and JCA-BM8060 (JEOL Ltd) biochemistry analyzers with Sysmex, Nittobo Medical, and LSI Medience reagents using standardized procedures and fresh samples. FPG was determined using a glucose glucokinase assay (LSI Medience Corp) and HbA1c concentrations were measured by LSI Medience using an automated biochemical analyzer JCA-BM9130 and JCA-BM9030 (JEOL Ltd). WBC counts were determined by flow cytometry as part of a full blood count. RBC and platelet counts were determined by electrical resistance detection. Hb concentration was determined by the SLS-Hb method, and Ht was measured by erythrocyte pulse peak detection. A clinician evaluated the safety of the supplementation based on these results and adverse events.

### Statistical analysis

Data are summarized as the means and SDs. Statistical significance of differences between the l-Ser + EPA group and the placebo group was assessed by ANCOVA using the initial scores at baseline as covariates at each time point, at a significance level of 5%. All statistical analyses were performed using IBM SPSS Statistics Ver. 24 or R version 3.5.0 (R Foundation) (36).

The statistical analysis was predetermined before key opening. The primary decision was planned in advance to be conducted based on the total scores of JLEQ and JKOM at week 8, and these 2 outcomes were tested by a fixed sequence procedure; the statistical significance test for JKOM score was conducted only when a significant improvement in the JLEQ score was observed. Thus, the type I error was controlled under the α = 5% level regarding the primary outcome. For other outcome measures, such as JLEQ and JKOM scores at other time points, subdomains of JLEQ and JKOM, JOA score, BPI, and EQ-5D-5L, statistical multiplicity was not considered because these do not affect the primary decision.

## Results

### Participants and compliance

We enrolled 60 participants in both groups (Figure 1). There were no dropouts, and all participants completed the study protocol; thus, the full analysis set included all study participants. The compliance rate in this study was high, such that >95% of capsules were consumed throughout the study in both groups. Mean age, height, weight, BMI, JLEQ, JKOM, and JOA scores at baseline are listed in **Table 1**. Fifty-one participants (active compared with placebo: 22 compared with 29) had pain in multiple body sites, such as the neck and shoulders, in addition to LBP and knee pain.

**TABLE 1:** General characteristics of the study participants in the l-serine (l-Ser) + EPA supplementation group and the placebo group at week 0^1^

	l-Ser + EPA (*n* = 60)	Placebo (*n* = 60)
Participants men/women (menopause)	18/42 (5)	18/42 (6)
Age, y	40.3 ± 11.0	41.4 ± 10.9
Height, cm	163 ± 9	163 ± 9
Weight, kg	59.1 ± 9.3	60.6 ± 13.0
BMI, kg/m^2^	22.3 ± 3.1	22.7 ± 3.2
JLEQ-I^2^	42.9 ± 15.7	41.6 ± 16.0
JLEQ score^3^	28.4 ± 13.4	27.3 ± 12.1
JKOM-I^4^	34.8 ± 17.4	31.3 ± 15.6
JKOM score^5^	18.2 ± 9.3	18.4 ± 7.9
JOA	21.4 ± 2.6	21.8 ± 2.2

^1^Mean age, height, weight, BMI, and JLEQ, JKOM, and JOA scores at baseline are demonstrated. Data are expressed as means ± SDs. JKOM, Japanese Knee Osteoarthritis Measure; JLEQ, Japan Low Back Pain Evaluation Questionnaire; JOA, Japanese Orthopedic Association; VAS, visual analogue scale.

^2^JLEQ-I: VAS for the degree of low-back pain.

^3^The JLEQ score is the sum of the JLEQ-II, -III, and -IV scores.

^4^JKOM-I: VAS for the degree of knee pain.

^5^The JKOM score is the sum of the JKOM-II, -III, -IV, and -V scores.

### LBP relief

**FIGURE 3:**
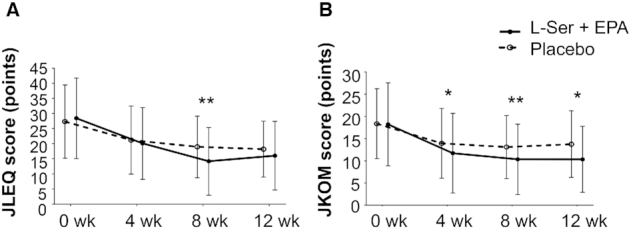
JLEQ (A) and JKOM (B) scores in the l-serine (l-Ser) + EPA supplementation group and the placebo group at weeks 0, 4, 8, and 12. Changes in JLEQ score (A) and JKOM score (B) from all study participants at weeks 0, 4, 8, and 12. The JLEQ score is the sum of the JLEQ-II, -III, and -IV scores, and the JKOM score is the sum of the JKOM-II, -III, -IV, and -V scores. Data are expressed as the means ± SDs, *n* = 60 in each group. ANCOVA using the baseline score as a covariate was performed to detect differences between the 2 groups by each time point. ^*, **^Significantly different from placebo group: *P < 0.05, **P < 0.01. JKOM, Japanese Knee Osteoarthritis Measure; JLEQ, Japan Low Back Pain Evaluation Questionnaire.

**Figure 3**A and **Supplemental Table 1** show the JLEQ scores, evaluating the issues regarding LBP. **Supplemental Table 2** presents the prevalence of participants with each issue in the JLEQ questionnaire. JLEQ scores decreased in both groups at weeks 4, 8, and 12 compared with baseline. As a primary outcome, at week 8, ANCOVA using the baseline scores as a covariate demonstrated significantly lower scores in the l-Ser + EPA group compared with the placebo group. Additionally, at week 8, a significant decrease in all 4 domains (I: VAS for the degree of LBP; II: LBP related to activities of daily living over the last several days; III: problems due to LBP over the last several days; and IV: health and psychological condition in the last month) was detected in the l-Ser + EPA group compared with the placebo group. The JOA score was not significantly different between the 2 groups.

### Knee pain relief

Figure 3B and Supplemental Table 1 show the JKOM scores, evaluating issues regarding knee pain. **Supplemental Table 3** demonstrates the prevalence of participants with each issue in the JKOM questionnaire. Although JKOM scores decreased in both groups at weeks 4, 8, and 12 compared with baseline, ANCOVA using the baseline scores as a covariate demonstrated significantly lower scores in the l-Ser + EPA group compared with the placebo group at week 8 as the primary outcome. In addition, significantly lower scores in the l-Ser + EPA group compared with the placebo group were observed at weeks 4 and 12.

Additionally, a significant decrease in the following 2 domains (I: VAS for the degree of knee pain; and II: pain and stiffness in the knee over the last several days) was detected in the l-Ser + EPA group compared with the placebo group at week 8. Interestingly, significant differences between these 2 groups were also detected in the posttreatment observation period at week 12 in the following domains: I: VAS for the degree of knee pain; II: pain and stiffness in the knee over the last several days; III: problems in daily life due to knee pain over the last several days; and IV and V: usual activities in the last month and general health status in the last month.

### QOL and overall pain relief

On the BPI, which evaluates the intensity of overall pain and the impact of pain on daily life, both groups showed significant improvement in all parameters from baseline to weeks 4, 8, and 12 (**Table 2**). ANCOVA demonstrated significant improvement in the l-Ser + EPA group compared with the placebo group at week 4 on BPI1: pain at its worst in the last 24 h; BPI3: pain on average; and BPI5D: intensity of the pain on moving. Similarly, at week 8, the BPI1, BPI3, and BPI5D scores were significantly lower in the l-Ser + EPA group than in the placebo group. In the posttreatment observation period at week 12, significant differences in BPI1 and BPI3 were not detected, whereas a significant decrease in BPI5D remained. **Supplemental Table 4** shows the improvement in the health-related QOL based on EQ-5D-5L due to l-Ser + EPA supplementation, at week 8.

**TABLE 2:** Brief Pain Inventory (BPI) results of the all study participants in the l-serine (l-Ser) + EPA supplementation group and the placebo group at weeks 0, 4, 8, and 12

	Baseline		Week 4			Week 8			Posttreatment observation period: week 12		
	l-Ser + EPA	Placebo	l-Ser + EPA	Placebo	*P* value^2^	l-Ser + EPA	Placebo	*P* value^2^	l-Ser + EPA	Placebo	*P* value^2^
BPI1: pain at its worst in the last 24 h	4.13 ± 1.63	4.12 ± 1.33	2.92 ± 1.51	3.35 ± 1.27	0.028*	2.35 ± 1.19	2.90 ± 1.19	0.004**	2.9 ± 1.5	3.2 ± 1.3	0.169
BPI2: pain at its least in the last 24 h	1.38 ± 1.04	1.27 ± 0.92	0.83 ± 0.83	0.88 ± 1.01	0.415	0.60 ± 0.74	0.65 ± 0.88	0.467	0.6 ± 1.0	0.6 ± 0.9	0.793
BPI3: pain on average	3.03 ± 1.39	2.77 ± 1.16	1.98 ± 1.16	2.22 ± 1.09	0.020*	1.58 ± 0.98	1.87 ± 0.97	0.019*	2.0 ± 1.1	2.0 ± 1.0	0.493
BPI4: pain right now	2.80 ± 1.55	2.62 ± 1.57	1.57 ± 1.35	1.70 ± 1.27	0.281	1.10 ± 1.12	1.33 ± 0.97	0.104	1.1 ± 1.3	1.4 ± 1.3	0.251
BPI5A: intensity of pain: lying	2.08 ± 1.63	2.05 ± 1.42	1.30 ± 1.28	1.30 ± 1.31	0.931	1.00 ± 1.19	1.08 ± 1.25	0.608	0.8 ± 1.4	0.9 ± 1.3	0.788
BPI5B: intensity of pain: sitting	2.75 ± 1.71	2.53 ± 1.59	1.83 ± 1.36	1.78 ± 1.33	0.797	1.33 ± 1.27	1.52 ± 1.31	0.197	1.7 ± 1.7	1.4 ± 1.3	0.413
BPI5C: intensity of pain: standing	3.28 ± 1.79	3.03 ± 1.57	1.93 ± 1.40	1.93 ± 1.30	0.512	1.53 ± 1.28	1.82 ± 1.41	0.069	2.0 ± 1.6	2.0 ± 1.5	0.664
BPI5D: intensity of pain: moving	3.60 ± 1.68	3.33 ± 1.66	2.22 ± 1.39	2.72 ± 1.33	0.001**	1.77 ± 1.25	2.43 ± 1.31	0.001**	2.1 ± 1.6	2.8 ± 1.6	0.007**

^1^BPI scores from all study participants at weeks 0, 4, 8 (dosing period), and 12 (posttreatment observation period). The BPI scores based on 8 questions are shown. Data are expressed as the means ± SDs, *n* = 60 in each group.

2 *P* values were obtained from ANCOVA between groups by each time point, using the scores at baseline as covariates. ^*,**^Significant difference between supplementation and placebo groups: *P < 0.05, **P < 0.01.

To evaluate the effects of l-Ser and EPA on multiple pain sites, an additional subgroup analysis was conducted on a study subset of 51 participants (active compared with placebo: 22 compared with 29) who had pain in multiple body sites, such as the neck and shoulders, in addition to LBP and knee pain. The results of the subgroup analysis of the 51 participants are shown in **Supplemental Table 5**. ANCOVA demonstrated a significant improvement in the l-Ser + EPA group compared with the placebo group at week 4 on BPI1: pain at its worst in the last 24 h; BPI3: pain at its worst in the last 24 h; BPI4: pain right now; BPI5B: intensity of pain when sitting; and BPI5D: intensity of pain when moving. Similarly, at week 8, the BPI1, BPI3, BPI4, BPI5B, and BPI5D scores were significantly lower in the l-Ser + EPA group than in the placebo group.

### Safety evaluations

No adverse events, including blood and urine laboratory test results, were observed. The assessment of safety was made through medical interviews with each participant, and no issues were reported. **Supplemental Table 6** shows the results of blood and urine laboratory tests.

## Discussion

This randomized, double-blind, placebo-controlled, parallel-group study examined the effect of oral l-Ser + EPA (594 mg and 149 mg daily, respectively) supplementation for 8 wk on back and knee pain intensity in healthy adults with LBP and knee pain. As the primary outcomes of the study, LBP, as assessed by JLEQ, and knee pain, as assessed by JKOM, were both significantly improved by l-Ser and EPA supplementation at week 8. The ANCOVA of JLEQ demonstrated significantly lower scores at week 8, assessed by both total scoring and scoring for each domain. The ANCOVA of JKOM demonstrated significantly lower scores at all time points: weeks 4, 8, and 12. The QOL related to pain was also significantly improved at all time points, as demonstrated by the BPI scores at weeks 4, 8, and 12. As demonstrated in a subgroup analysis of the 51 participants who had pain in multiple body sites, the l-Ser + EPA supplementation also relieved the symptoms of participants who had multiple-site pain. We also observed a very high compliance rate as well as study completion rate, and adverse events were not observed in either of the groups. These results suggest that l-Ser and EPA supplementation is both safe and effective in improving pain and QOL in adults with chronic and nonspecific pain in multiple body sites, including LBP and knee pain.

In this study, JOA was also used for the evaluation of LBP, but the scores were not significantly different between the 2 groups. Compared with JLEQ, which is a patient-reported outcome that focuses more on QOL aspects of pain, JOA is a physician-reported measure that has been widely used to evaluate the clinical results of various surgical and nonsurgical interventions performed on patients with LBP. Therefore, the characteristics of the healthy adult study population could have been related to the limitations in JOA results.

LBP is considered chronic if it has been present for >3 mo. The etiology of chronic LBP is, in most cases (≤85%), unknown or nonspecific, whereas specific causes (specific spinal pathology and neuropathic/radicular disorders) are uncommon (2). Often, the condition or injury that triggered the pain can be healed and undetectable, but the pain can continue. Recent studies have investigated the potential relation between central sensitization and chronic pain disorders. Enhanced excitability in the central nervous system is an important phenomenon observed in people with chronic LBP (37, 38) and also occurs in various other pain conditions (39, 40). Given the increasing evidence supporting the clinical significance of central sensitization in chronic pain, effective methods designed to normalize pain physiology are required.

There have been several clinical studies regarding the oral administration of l-Ser. Oral l-Ser administration has shown effectiveness in patients with hereditary sensory autonomic neuropathy type I, who suffer a debilitating, progressive disorder of peripheral nerves that results in sensory loss and neuropathic pain (41–43). Additionally, an exploratory study suggested that l-Ser can slow disease progression in patients with amyotrophic lateral sclerosis (44, 45). Kiya et al. (25) reported that the administration of l-Ser improved peripheral hyperalgesia in a paclitaxel-induced hyperalgesia model, especially focusing on the function of l-Ser in the DRG.

In vitro studies have also demonstrated that l-Ser is essential for maintaining normal functions of the nervous system. Savoca et al. (46) demonstrated that l-Ser is an important factor for the morphological differentiation of neurons in vitro through the observation of marked effects on dendritogenesis and axon length when l-Ser was added to developing neurons. Additionally, a study conducted in an animal model of brain injury revealed that l-Ser plays a role in inducing the proliferation and differentiation of neural stem cells and promoting the repair of nerve injury (47). Additionally, whereas demyelination contributes to the development of neuropathic pain by disrupting the molecular and structural features of nerve fibers (12), l-Ser is an important component for neural cell membrane and myelin formation (11). These findings indicate that l-Ser is an essential factor for neural cells to function properly.

**FIGURE 4:**
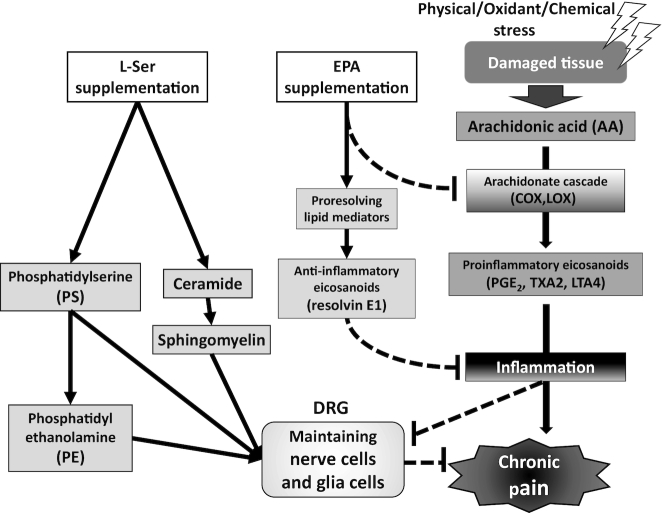
Possible mechanism of pain relief from combinatory administration of l-serine (l-Ser) and EPA in relieving chronic low-back pain and knee pain. l-Ser provides essential components in support of damaged nerve cells and glial cells, and thus plays an important role in maintaining normal functions of the nervous system. EPA competes with arachidonic acid, thereby suppressing the production of eicosanoids and inflammation. EPA also produces the anti-inflammatory lipid mediator resolvin E1. We hypothesize that l-Ser and EPA synergistically work in the dorsal root ganglion (DRG), by simultaneously providing necessary components to maintain nerve function and mitigating local inflammation in the DRG. COX, cyclooxygenase; LOX, lipoxygenase; LTA4, leukotriene A4; TXA2, thromboxane A2.

EPA competes with arachidonic acid, thereby suppressing the production of eicosanoids and inflammation. It has also been reported that the anti-inflammatory lipid mediator resolvin E1 is produced from EPA via an intracellular biosynthetic pathway when activated neutrophils adhere to vascular endothelial cells in local inflammation. In addition to its anti-inflammatory properties, resolvin E1 suppresses NMDA hyperfunction caused by transient activity of receptor potential cation channel subfamily V member 1 and TNF-α by inhibiting extracellular signal-regulated kinase signaling in spinal dorsal horn neurons. Through this mechanism, pain and hyperalgesia in response to heat and mechanical stimuli are alleviated (20–22). These reports suggest that EPA is useful for the treatment of neuropathic pain triggered by inflammation.

l-Ser could support neuronal function in damaged nerve fibers by providing essential components required for normal neuronal function. EPA could exert anti-inflammatory properties and reduce local inflammation, thereby suppressing pain signals from neuronal fibers (**Figure 4**). Table 2 demonstrates that l-Ser and EPA supplementation alleviated overall pain, and Supplemental Table 5 demonstrates that the l-Ser + EPA supplementation also relieved the symptoms of participants who had multiple-site pain such as the neck and shoulders, in addition to LBP and knee pain, suggesting that l-Ser and EPA might act on similar pathways in these multiple sites and improve neuropathic pain. l-Ser and EPA might synergistically work in the DRG, by providing necessary components for nerve function and mitigating local inflammation. Additional studies in the future are necessary to clarify the detailed mechanism. The results from the posttreatment observation period in the current study demonstrated a significant difference in the degree of knee pain between the l-Ser + EPA group and the placebo group, indicating that l-Ser and EPA provide continued effects even after supplementation has ceased. Compared with other pain management methods that are often used for temporary pain relief, l-Ser and EPA could have the potential to provide more lasting effects.

There were several limitations in this study. Firstly, evaluation during the l-Ser and EPA ingestion period was performed only at weeks 4 and 8. We do not have data regarding effects on the pain scores with shorter-term and longer-term ingestion. Secondly, only a limited subgroup analysis was conducted in this study to examine the relation between background information and improvement in outcome scores. In particular, because the l-Ser concentration decreases with aging (14), stratification analysis using biomarkers for efficacy prediction is also important. Thirdly, we did not collect dietary records in this study. In the future, dietary intake should be assessed for potential deficiencies or excess intake of nutrients such as l-Ser and EPA, which could provide additional insight to the mechanistic rationale.

In conclusion, this randomized, double-blind, placebo-controlled, parallel-group study demonstrated that supplementation with l-Ser and EPA improved the pain scores of a generally healthy adult population with pain in at least the low back and knee for ≥3 mo. The compliance rate was high throughout the study, and no adverse events (including abnormal blood and urine laboratory test results) were observed. To our knowledge, this is the first study to report beneficial effects of the combinatory administration of l-Ser and EPA in improving such pain. Although further research is needed to clarify the underlying mechanism related to this effect, l-Ser and EPA supplementation could provide effective pain management and improve QOL in people with chronic LBP and knee pain.

## Supplementary Material

nxaa156_Supplemental_File

## Notes

Ajinomoto Co, Inc, funded the study and participated in the study design, implementation, analysis, and interpretation of the data.

Author disclosures: IS, AY, M Takeshita, YS, KS, NN, M Takada, MH, TM, and KN are employees of Ajinomoto Co, Inc. YK conducted research funded by Ajinomoto Co, Inc.

Supplemental Tables 1–6 are available from the “Supplementary data” link in the online posting of the article and from the same link in the online table of contents at https://academic.oup.com/jn/.

Abbreviation used: BPI, Brief Pain Inventory; DRG, dorsal root ganglion; EQ-5D-5L, QOL assessment by EuroQOL 5 Dimensions 5-level; FPG, fasting plasma glucose level; Hb, hemoglobin; HbA1c, glycated hemoglobin; Ht, hematocrit; JKOM, Japanese Knee Osteoarthritis Measure; JLEQ, Japan Low Back Pain Evaluation Questionnaire; JOA, Japanese Orthopedic Association; LBP, low-back pain; l-Ser, l-serine; NMDA, *N*-methyl-d-aspartic acid; QOL, quality of life; WBC, white blood cell.
