# Supplementary material for: l-Serine and EPA Relieve Chronic Low-Back and Knee Pain in Adults: A Randomized, Double-Blind, Placebo-Controlled Trial
Source: J Nutr. 2020 Jun 10;150(9):2278–86. doi: 10.1093/jn/nxaa156 (PMC7467851; doi:10.1093/jn/nxaa156)
Supplement: nxaa156_Supplemental_File [file nxaa156_supplemental_file.docx]

# **L-Serine and eicosapentaenoic acid relieve chronic low-back and knee pain in adults: a randomized, double-blind, placebo-controlled trial. Sasahara et al. Online supplementary material**

**Supplementary Table 1. Low-back and knee pain scores of the all study participants in the L-Ser and EPA supplementation group and the placebo group at week 0, 4, 8, and 12**

|  | Baseline | | Week 4 | | | Week 8 | | | Post-treatment observation period;  Week 12 | | |
| --- | --- | --- | --- | --- | --- | --- | --- | --- | --- | --- | --- |
|  | L-Ser+EPA | Placebo | L-Ser+EPA | Placebo | *p* value^1^ | L-Ser+EPA | Placebo | *p* value^1^ | L-Ser+EPA | Placebo | *p* value^1^ |
| JLEQ score | 28.4 ± 13.4 | 27.3 ± 12.1 | 20.1 ± 11.9 | 21.2 ± 11.3 | 0.231 | 14.2 ± 11.2 | 19.0 ± 10.2 | <0.001** | 16.0 ± 11.4 | 18.2 ± 9.3 | 0.056 |
| JLEQ-I | 42.9 ± 15.7 | 41.6 ± 16.0 | 29.9 ± 15.6 | 32.1 ± 17.7 | 0.296 | 23.5 ± 15.8 | 31.4 ± 15.2 | <0.001** | 28.9 ± 14.9 | 31.5 ± 13.7 | 0.141 |
| JLEQ-II | 9.0 ± 3.1 | 9.1 ± 3.5 | 7.1 ± 3.0 | 7.5 ± 3.3 | 0.525 | 5.4 ± 3.1 | 6.8 ± 3.3 | 0.005** | 5.8 ± 3.1 | 6.6 ±3.1 | 0.068 |
| JLEQ-III | 15.8 ± 8.6 | 14.3 ± 7.3 | 10.7 ± 7.4 | 11.2 ± 7.0 | 0.150 | 7.5 ± 7.0 | 9.7 ± 6.1 | 0.003** | 8.7 ± 7.2 | 9.2 ± 5.7 | 0.149 |
| JLEQ-IV | 3.6 ± 3.2 | 3.9 ± 2.7 | 2.3 ± 3.0 | 2.6 ± 2.1 | 0.630 | 1.3 ± 1.9 | 2.5 ± 2.0 | <0.001** | 1.6 ± 1.9 | 2.3 ± 1.6 | 0.013* |
| JOA score | 21.4 ± 2.6 | 21.8 ± 2.2 | 23.4 ± 2.2 | 22.9 ± 2.2 | 0.069 | 24.2 ± 1.8 | 24.0 ± 2.1 | 0.321 | 23.5 ± 2.2 | 23.8 ± 1.7 | 0.792 |
| JKOM score | 18.2 ± 9.3 | 18.4 ± 7.9 | 11.7 ± 9.0 | 13.9 ± 7.9 | 0.040* | 10.4 ± 7.9 | 13.1 ± 7.1 | 0.006** | 10.3 ± 7.4 | 13.8 ± 7.5 | 0.001** |
| JKOM-I | 34.8 ± 17.4 | 31.3 ± 15.6 | 22.3 ± 16.4 | 24.7 ± 13.1 | 0.068 | 18.2 ± 12.7 | 25.2 ± 15.3 | <0.001** | 20.0 ± 14.3 | 25.4 ± 15.4 | 0.005** |
| JKOM-II | 7.5 ± 3.4 | 7.4 ± 3.3 | 4.8 ± 2.3 | 5.9 ± 3.5 | 0.013* | 4.2 ± 2.9 | 5.6 ± 3.2 | 0.001** | 4.1 ± 2.8 | 5.5 ± 3.1 | <0.001** |
| JKOM-III | 5.3 ± 3.7 | 5.4 ± 3.6 | 2.8 ± 3.5 | 3.3 ± 3.2 | 0.340 | 2.5 ± 3.1 | 3.2 ± 3.2 | 0.105 | 2.6 ± 2.8 | 3.5 ± 3.4 | 0.039* |
| JKOM-IV & V | 5.5 ± 3.1 | 5.6 ± 2.2 | 4.1 ± 3.1 | 4.8 ± 2.3 | 0.098 | 3.7 ± 2.8 | 4.3 ± 1.9 | 0.077 | 3.7 ± 2.5 | 4.7 ± 2.1 | 0.006** |

^1^P-values were obtained from analysis of covariance between groups by each time point, using the scores at baseline as covariates. **p*<0.05, ***p*<0.01.

Low-back and knee pain scores from all study participants at week 0, 4, 8 (dosing period), and 12 (post-treatment observation period). Data are expressed as the means ± SDs, *n* = 60 in each group. JLEQ-I: VAS for the degree of low-back pain (LBP); JLEQ-II: LBP related to activities of daily living over the last several days; JLEQ-III: problems due to LBP over the last several days; JLEQ-IV: health and psychological condition in the last month. The JLEQ score is the sum of the JLEQ-II, III, and IV scores. JKOM-I: VAS for the degree of knee pain; JKOM-II: pain and stiffness in the knee over the last several days; JKOM-III: problems in daily life due to knee pain over the last several days; and JKOM- IV & V: usual activities in the last month and general health status in the last month. The JKOM score is the sum of the JKOM-II, III, IV, and V scores.

**Supplementary Table 2. Prevalence of participants with each issue in the JLEQ questionnaire. L-Ser and EPA group.**

|  |  | n | % |  |
| --- | --- | --- | --- | --- |
| JLEQ-II | JLEQ1 | 56 | 43 | Over the last several days, what type of pain have you felt in your lower back when lying on your back? |
|  | JLEQ2 | 58 | 53 | Over the last several days, what type of pain have you felt in your lower back on waking in the morning and moving about? |
|  | JLEQ3 | 53 | 62 | Over the last several days, what type of pain have you felt in your lower back when sitting in a chair? |
|  | JLEQ4 | 53 | 49 | Over the last several days, what type of pain have you felt in your lower back when you have stood up or squatted? |
|  | JLEQ5 | 57 | 47 | Over the last several days, what type of pain have you felt in your lower back when standing? |
|  | JLEQ6 | 58 | 45 | Over the last several days, what type of pain have you felt in your lower back when bending over? |
|  | JLEQ7 | 56 | 48 | Over the last several days, what type of pain have you felt in your lower back when bending backward? |
| JLEQ-III | JLEQ8 | 60 | 58 | Over the last several days, to what extent has it been painful to maintain the same posture? |
|  | JLEQ9 | 40 | 60 | Over the last several days, to what extent has it been difficult due to lower back pain to turn over during sleep? |
|  | JLEQ10 | 47 | 68 | Over the last several days, to what extent has it been difficult due to lower back pain to get up in the morning? |
|  | JLEQ11 | 47 | 49 | For the last several days, to what extent has it been difficult due to lower back pain to move? |
|  | JLEQ12 | 33 | 67 | For the last several days, to what extent has it been difficult due to lower back pain to get up from a chair or Western style toilet? |
|  | JLEQ13 | 37 | 59 | For the last several days, to what extent has it been difficult due to lower back pain to go up and down stairs? |
|  | JLEQ14 | 34 | 62 | For the last several days, to what extent has it been difficult due to lower back pain to put on socks or stockings? |
|  | JLEQ15 | 30 | 57 | For the last several days, to what extent has it been difficult due to lower back pain to put on and take off trousers and slacks? |
|  | JLEQ16 | 48 | 54 | For the last several days, to what extent has it been difficult due to lower back pain to lift from the floor objects weighing 3-4 kilograms（2 standard sake bottles or 2 PET bottles each containing 2 liters）? |
|  | JLEQ17 | 54 | 52 | For the last several days, to what extent has it been difficult due to lower back pain to twist around and pick up something from behind? |
|  | JLEQ18 | 30 | 53 | For the last several days, to what extent have you been restricted due to lower back pain in walking outside? |
|  | JLEQ19 | 39 | 69 | For the last several days, to what extent have simple tasks and housework（cleaning, preparing meals, etc） been difficult due to lower back pain? |
|  | JLEQ20 | 55 | 64 | For the last several days, to what extent have load-bearing tasks and housework （carrying heavy objects, and cleaning in the yard, etc） been difficult due to lower back pain? |
|  | JLEQ21 | 51 | 71 | For the last several days, have you wanted to lie down and rest due to lower back pain? |
|  | JLEQ22 | 43 | 74 | For the last several days, have you wanted to skip work or school, or normal tasks or housework due to lower back pain? |
|  | JLEQ23 | 18 | 72 | For the last several days have you been unable to sleep well at night due to lower back pain? |
|  | JLEQ24 | 28 | 75 | Looking at the condition of your lower back for the last several days, do you think it would be difficult to leave home for an extended period? |
| JLEQ-IV | JLEQ25 | 17 | 88 | In the last month, have you refrained from going out into the neighborhood due to lower back pain? |
|  | JLEQ26 | 21 | 81 | In the last month, have you been restricted from performing normal activities （meeting friends, engaging in a sport, engaging in activities and hobbies, etc.） due to lower back pain? |
|  | JLEQ27 | 8 | 88 | In the last month, have you taken days off from work or school other than weekends/holidays or rested from normally performed housework due to lower back pain? |
|  | JLEQ28 | 39 | 69 | In the last month, have you ever felt unwell due to lower back pain? |
|  | JLEQ29 | 43 | 60 | In the last month, do you think your lower back pain has affected your mental health? |
|  | JLEQ30 | 48 | 54 | In the last month, do you think your lower back pain has adversely affected your general well being? |

Original questions are written in Japanese.

JLEQ-II (JLEQ1 through JLEQ7): LBP related to activities of daily living over the last several days; JLEQ-III (JLEQ8 through JLEQ24): problems due to LBP over the last several days; JLEQ-IV (JLEQ25 through JLEQ30): health and psychological condition in the last month. These 30 questions are each ranked on a 5-point scale from no impairment (0 points) to serious impairment (4 points). The “n” indicates the number of participants out of 60 who ranked at a score of 1 or higher for the question at baseline. The “%” indicates the ratio of participants who decreased the rank from baseline to week 8.

**Supplementary Table 3. Prevalence of participants with each issue in the JKOM questionnaire. L-Ser and EPA group.**

|  |  | n | % |  |
| --- | --- | --- | --- | --- |
| JKOM-II | JKOM1 | 45 | 64 | Do you feel stiffness in your knees when you wake up in the morning? |
|  | JKOM2 | 52 | 48 | Do you feel pain in your knees when you wake up in the morning? |
|  | JKOM3 | 12 | 100 | How often do you wake up in the night because of pain in your knees? |
|  | JKOM4 | 41 | 54 | Do you have pain in your knees when you walk on a flat surface? |
|  | JKOM5 | 56 | 48 | Do you have pain in your knees when ascending stairs? |
|  | JKOM6 | 56 | 57 | Do you have pain in your knees when descending stairs? |
|  | JKOM7 | 59 | 46 | Do you have pain in your knees when bending to the floor or standing up? |
|  | JKOM8 | 51 | 53 | Do you have pain in your knees when standing? |
| JKOM-III | JKOM9 | 59 | 63 | How difficult is ascending or descending stairs? |
|  | JKOM10 | 59 | 56 | How difficult is bending to the floor or standing up? |
|  | JKOM11 | 24 | 54 | How difficult is standing up from sitting on a western style toilet? |
|  | JKOM12 | 23 | 48 | How difficult is wearing pants, skirts, and underwear? |
|  | JKOM13 | 20 | 45 | How difficult is putting on socks? |
|  | JKOM14 | 19 | 58 | How long can you walk on a flat surface without taking a rest? [More than 30 min, about 15 min, around my house, can hardly  walk] |
|  | JKOM15 | 0 | NA | Have you been using a walking stick (cane) recently? [Not at all, hardly, sometimes, often, always] |
|  | JKOM16 | 16 | 75 | How difficult is shopping for daily necessities? [Not at all, a little, moderately, quite, extremely] |
|  | JKOM17 | 20 | 65 | How difficult is doing light housework (cleaning the dining room after eating, etc.)? [Not at all, a little, moderately, quite,  extremely] |
|  | JKOM18 | 37 | 65 | How difficult is doing heavy housework (using the vacuum cleaner, etc.)? [Not at all, a little, moderately, quite, extremely] |
| JKOM-IV&V | JKOM19 | 55 | 35 | Have you gone to an event or to a department store during the last one month? [More than 2-3 times a week, about once a week,  about once every 2 weeks, once a month, not at all] |
|  | JKOM20 | 19 | 68 | Were things that you usually do (some kind of lesson, meeting friends, etc.) difficult because of knee pain during the last one  month? [Not at all, a little, moderately, quite, extremely] |
|  | JKOM21 | 13 | 62 | Did you limit doing things you usually do because of knee pain during the last one month? [Not at all, a little, moderately, quite,　didn’t do them (things you do usually) at all] |
|  | JKOM22 | 9 | 56 | Did you despair of going outside somewhere close because of knee pain during the last one month? [Not at all, hardly, sometimes,  often, didn’t go outside (close)] |
|  | JKOM23 | 11 | 82 | Did you despair of going outside somewhere far because of knee pain during the last one month? [Not at all, hardly, sometimes,　often, didn’t go outside (far)] |
|  | JKOM24 | 58 | 41 | Do you think your health during the last one month is average? [I really think so, I think so, I don’t know, I don’t think so, I　don’t think so at all] |
|  | JKOM25 | 44 | 48 | Do you think that knee pain has been affecting your health badly during the last one month? [It isn’t affecting it at all, It is affecting　it a little, It is affecting it moderately, It is affecting it significantly, It is affecting it greatly] |

Original questions are written in Japanese.

JKOM-II (JKOM1 through JKOM8): pain and stiffness in the knee over the last several days. JKOM-III (JKOM9 through JKOM18): problems in daily life due to knee pain over the last several days. JKOM-IV&V (JKOM19 through JKOM25): the usual activities in the last month and general health status in the last month. These 25 questions are each ranked on a 5-point scale from no impairment (0 points) to serious impairment (4 points). The “n” indicates the number of participants out of 60 who ranked at a score of 1 or higher for the question at baseline. The “%” indicates the ratio of participants who decreased the rank from baseline to week 8.

**Supplementary Table 4. Health-related QOL based on the EQ-5D-5L score of the all study participants in the L-Ser and EPA supplementation group and the placebo group at week 0, 4, 8, and 12**

|  | Baseline | | Week 4 | | | Week 8 | | | Post-treatment observation period;  Week 12 | | |
| --- | --- | --- | --- | --- | --- | --- | --- | --- | --- | --- | --- |
|  | L-Ser+EPA | Placebo | L-Ser+EPA | Placebo | *p* value^1^ | L-Ser+EPA | Placebo | *p* value^1^ | L-Ser+EPA | Placebo | *p* value^1^ |
| EQ-5D-5L score | 0.79 ± 0.10 | 0.80 ± 0.11 | 0.85 ± 0.10 | 0.85 ± 0.10 | 0.942 | 0.90 ± 0.09 | 0.87 ± 0.08 | 0.047* | 0.87 ± 0.09 | 0.88 ± 0.08 | 0.694 |

Data are expressed as the means ± SDs, *n* = 60 in each group. ^1^P-values were obtained from analysis of covariance between groups by each time point, using the scores at baseline as covariates. **p*<0.05, ***p*<0.01.

EQ-5D QOL scores increased from baseline to weeks 4, 8, and 12 in both groups. At week 8, the analysis of covariance demonstrated a significant improvement in the L-Ser and EPA group compared with the placebo group.

**Supplementary Table 5. Brief Pain Inventory (BPI) subgroup analysis of participants who had pain in multiple body sites in the L-Ser and EPA supplementation group and the placebo group at week 0, 4, 8, and 12**

|  | Baseline | | Week 4 | | | Week 8 | | | Post-treatment observation period;  Week 12 | | |
| --- | --- | --- | --- | --- | --- | --- | --- | --- | --- | --- | --- |
|  | L-Ser+EPA | Placebo | L-Ser+EPA | Placebo | *p* value^1^ | L-Ser+EPA | Placebo | *p* value^1^ | L-Ser+EPA | Placebo | *p* value^1^ |
| BPI1: Pain at its worst in the last 24 hours | 3.82 ± 1.74 | 4.07 ± 1.41 | 2.41 ± 1.44 | 3.48 ± 1.43 | 0.004** | 2.09 ± 1.02 | 2.86 ± 1.38 | 0.023* | 2.50 ±1.60 | 3.31 ± 1.37 | 0.070 |
| BPI2: Pain at its least in the last 24 hours | 1.05 ± 1.00 | 1.28 ± 1.10 | 0.64 ± 0.73 | 1.07 ± 1.28 | 0.247 | 0.45 ± 0.60 | 0.72 ± 1.10 | 0.475 | 0.59 ± 0.91 | 0.62 ±0.94 | 0.734 |
| BPI3: Pain on average | 2.68 ± 1.49 | 2.83 ± 1.26 | 1.59 ± 1.10 | 2.41 ± 1.32 | 0.011* | 1.27 ± 0.88 | 1.93 ±1.25 | 0.025* | 1.82 ±1.26 | 2.10 ± 1.11 | 0.450 |
| BPI4: Pain right now | 2.68 ± 1.67 | 2.66 ± 1.72 | 1.27 ± 1.08 | 1.93 ± 1.41 | 0.028* | 0.82 ±0.91 | 1.38 ±1.18 | 0.043* | 1.09 ± 1.41 | 1.45 ±1.30 | 0.302 |
| BPI5A: Intensity of pain: lying | 2.18 ± 1.74 | 2.31 ± 1.37 | 1.09 ± 1.11 | 1.72 ± 1.51 | 0.067 | 0.77 ± 0.92 | 1.34 ± 1.54 | 0.095 | 0.73 ± 1.55 | 1.03 ± 1.59 | 0.540 |
| BPI5B: Intensity of pain: sitting | 2.45 ± 1.82 | 2.86 ± 1.68 | 1.50 ± 1.06 | 2.31 ± 1.49 | 0.038* | 1.05 ± 0.95 | 1.83 ± 1.44 | 0.043* | 1.50 ± 1.74 | 1.69 ± 1.54 | 0.917 |
| BPI5C: Intensity of pain: standing | 2.77 ± 1.85 | 3.17 ± 1.61 | 1.55 ± 1.06 | 2.10 ± 1.54 | 0.238 | 1.27 ± 1.12 | 1.76 ± 1.55 | 0.363 | 1.77 ±1.60 | 2.10 ± 1.50 | 0.726 |
| BPI5D: Intensity of pain: moving | 3.18 ± 1.82 | 3.38 ± 1.76 | 1.82 ± 1.44 | 2.72 ± 1.46 | 0.009** | 1.50 ± 1.14 | 2.28 ± 1.46 | 0.030* | 2.00 ± 1.83 | 2.55 ± 1.82 | 0.313 |

Brief Pain Inventory (BPI) scores from all study participants at week 0, 4, 8 (dosing period), and 12 (post-treatment observation period). Data are expressed as the means ± SDs. Fifty-one participants (active vs placebo; 22 vs 29) who had pain in multiple body sites, such as the neck and shoulders, in addition to low-back and knee pain were included in the analysis. The BPI scores, consisting of 8 questions are demonstrated

^1^P-values were obtained from analysis of covariance between groups by each time point, using the scores at baseline as covariates. *p<0.05, **p<0.01.

**Supplementary Table 6. Safety evaluation data of the all study participants in the L-Ser and EPA supplementation group and the placebo group at week 0 and 8**

|  | Baseline | | Week 8 | |
| --- | --- | --- | --- | --- |
|  | L-Ser+EPA | Placebo | L-Ser+EPA | Placebo |
| SBP, mmHg | 119 ± 10 | 120 ± 11 | 119 ±11 | 120 ±12 |
| DBP, mmHg | 72 ± 7 | 73± 8 | 72 ± 8 | 73 ± 8 |
| Pulse rate, bpm | 70 ± 7 | 71 ± 8 | 71 ± 7 | 71 ± 7 |
| WBC, /mL | 5500 ± 1270 | 5420 ± 1260 | 5270 ± 1340 | 5340 ± 1290 |
| RBC, ×10^4^/mL | 466 ± 40 | 471 ± 46 | 463 ± 45 | 477 ± 50 |
| Hb, g/dL | 13.8 ± 1.4 | 14.1 ± 1.4 | 13.6 ± 1.7 | 14.2 ± 1.5 |
| Ht, % | 43.4 ± 3.9 | 44.0 ± 3.6 | 43.1 ± 4.4 | 44.4 ± 3.9 |
| PLT, ×10^4^/mL | 30.7 ± 6.7 | 29.8 ± 6.2 | 29.5 ± 7.0 | 29.1 ± 6.5 |
| TP, g/dL | 7.3 ± 0.5 | 7.2 ± 0.4 | 7.2 ± 0.4 | 7.2 ±0 .4 |
| Alb, g/dL | 4.4 ± 0.3 | 4.4 ± 0.3 | 4.4 ± 0.2 | 4.4 ± 0.3 |
| AST, U/L | 19.4 ± 5.1 | 19.2 ± 3.9 | 19.0 ± 6.4 | 20.6 ± 8.6 |
| ALT, U/L | 15.7 ± 8.6 | 16.5 ± 7.7 | 17.2 ± 16.2 | 18.8 ± 13.1 |
| LD, U/L | 173 ± 36 | 167 ± 26 | 171 ± 33 | 169 ± 29 |
| T-Bil, mg/dL | 0.8 ± 0.3 | 0.8 ± 0.3 | 0.7 ± 0.2 | 0.7 ± 0.2 |
| ALP, U/L | 183 ± 49 | 192 ± 45 | 184 ± 51 | 197 ± 54 |
| γ-GT, U/L | 21.8 ± 12.4 | 23.7 ± 17.2 | 21.9 ± 13.2 | 25.6 ± 25.6 |
| UN, mg/dL | 11.9 ± 3.0 | 12.4 ± 3.4 | 11.8 ± 3.1 | 12.3 ± 3.2 |
| Cr, mg/dL | 0.67 ± 0.13 | 0.66 ± 0.13 | 0.68 ± 0.13 | 0.67 ± 0.14 |
| UA, mg/dL | 4.7 ± 1.2 | 4.9 ± 1.2 | 4.7 ± 1.2 | 4.8 ± 1.4 |
| Na, mEq/L | 141 ± 2 | 141 ± 2 | 141 ± 2 | 141 ± 2 |
| Cl, mEq/L | 103 ± 2 | 104 ± 2 | 104 ± 2 | 104 ± 2 |
| K, mEq/L | 4.4 ± 0.3 | 4.3 ± 0.3 | 4.3 ± 0.3 | 4.3 ± 0.3 |
| T-Cho, mg/dL | 196 ± 33 | 201 ± 25 | 192 ± 35 | 202 ± 26 |
| LDL-Cho, mg/dL | 112 ± 28 | 119 ± 23 | 109 ± 31 | 118 ± 24 |
| HDL-Cho, mg/dL | 67.7 ± 15.9 | 65.2 ± 15.1 | 67.2 ± 15.4 | 65.0 ± 13.5 |
| TG, mg/dL | 80.2 ± 41.7 | 84.7 ± 45.1 | 80.4 ± 34.6 | 93.9 ± 52.0 |
| FPG, mg/dL | 82.5 ± 6.8 | 83.8 ± 7.4 | 82.6 ± 6.9 | 84.2 ± 8.6 |
| HbA1c, % | 5.3 ± 0.3 | 5.3 ± 0.3 | 5.4 ± 0.3 | 5.4 ± 0.3 |
|  |  |  |  |  |
| Urinary protein | 0.1 ± 0.2 | 0.0 ± 0.0 | 0.1 ± 0.3 | 0.0 ± 0.2 |
| Urinary glucose | 0.0 ± 0.0 | 0.0 ± 0.0 | 0.0 ± 0.0 | 0.0 ± 0.0 |
| Urinary occult blood | 0.2 ± 0.8 | 0.1 ± 0.4 | 0.3 ± 0.7 | 0.2 ± 0.9 |

The results of blood and urine laboratory tests of the all study participants in the L-Ser and EPA supplementation group and the placebo group at week 0 and 8

Data are expressed as the means ± SDs, *n* = 60 in each group.
